# Automated Segmentation Method for Low Field 3D Stomach MRI Using Transferred Learning Image Enhancement Network

**DOI:** 10.1155/2021/6679603

**Published:** 2021-02-11

**Authors:** Luguang Huang, Mengbin Li, Shuiping Gou, Xiaopeng Zhang, Kun Jiang

**Affiliations:** ^1^Xijing Hospital of the Fourth Military Medical University, Xian, Shaanxi, China; ^2^School of Artificial Intelligent, Xidian University, Xian, Shaanxi, China; ^3^Intelligent Medical Imaging Big Data Frontier Research Center, Xidian University, Xian, Shaanxi, China

## Abstract

Accurate segmentation of abdominal organs has always been a difficult problem, especially for organs with cavities. And MRI-guided radiotherapy is particularly attractive for abdominal targets compared with low CT contrast. But in the limit of radiotherapy environment, only low field MRI segmentation can be used for stomach location, tracking, and treatment planning. In clinical applications, the existing 3D segmentation network model is trained by the low field MRI, and the segmentation result cannot be used in radiotherapy plan since the bad segmentation performance. Another way is that historical high field intensity MR images are directly used for data expansion to network learning; there will be a domain shift problem. How to use different domain images to improve the segmentation accuracy of deep neural network? A 3D low field MRI stomach segmentation method based on transfer learning image enhancement is proposed in this paper. In this method, Cycle Generative Adversarial Network (CycleGAN) is used to construct and learn the mapping relationship between high and low field intensity MRI and to overcome domain shift. Then, the image generated by the high field intensity MRI through the CycleGAN network is with transferred information as the extended data. The low field MRI combines these extended datasets to form the training data for training the 3D Res-Unet segmentation network. Furthermore, the convolution layer, batch normalization layer, and Relu layer together were replaced with a residual module to relieve the gradient disappearance of the neural network. The experimental results show that the Dice coefficient is 2.5 percent better than the baseline method. The over segmentation and under segmentation are reduced by 0.7 and 5.5 percent, respectively. And the sensitivity is improved by 6.4 percent.

## 1. Introduction

Image-guided radiotherapy has become the mainstream of radiotherapy for gastric cancer, and it is very important to refer to the precise contour of target organs in the process of image-guided radiotherapy. CT has low contrast for soft tissue, so it is very difficult to locate and trace accurately abdominal organs. Dynamic Magnetic Resonance Imaging (dMRI) has the flexibility to image in the orientations most relevant to organ and tumor motion and for a prolonged duration without ionizing radiation. Therefore, MRI-guided radiotherapy is particularly attractive for abdominal targets. The normal MRI can provide a high spatial resolution anatomy and morphology proton distribution information of organs to get accurately tumor contour [1–3]. But it cannot meet the requirements of image-guided radiotherapy since high field interferes radiotherapy equipment. In order to realize the real-time MRI-guided radiotherapy, American ViewRay company developed the MRIdian system with a magnetic field intensity of 0.35 T, which makes the peak signal-to-noise ratio (PSNR) and spatial resolution of the images are very low.

At present, the low field MRI-guided radiotherapy is very few, which leads to the serious shortage of low field MR images. If high field MRI are directly used for data expansion training, domain shift will reduce greatly the segmentation performance of the existing deep learning model. Inspired by transfer learning and CycleGAN model, one way of meeting clinical application is to make high field intensity MRI transfer to low field intensity MRI to expand training data of the deep neural network [4].

Recently, many segmentation models based on UNet [5, 6] have been proposed in the last few years, like HDenseUNet [7], nnUnet res-Unet [8, 9], and LW-HCN, in which res-Unet achieved state-of-the-art performance on segmentation tasks in a different medical dataset. But these networks (Unets) are based on single domain segmentation task. And few reports were on the multidomain segmentation problem. To reduce the appearance gap cross-image modalities, a generative adversarial network (GAN) has been proposed to generate an image following a distribution. Nie et al. [10] introduced the GAN in medical image synthesis. Tanner et al. proposed a GAN for MR-CT deformable registration. Zhu et al. [11, 12] proposed the Cycle Generative Adversarial Network (CycleGAN) for the image translation from different domains. Compared with the GAN, it can be efficiently trained by the unpaired image data. This advantage can benefit to the cross-domain medical image registration. In particular, for the cross-modal medical images with big appearance and morphological gaps, CycleGAN can be introduced to map relieve domain shift.

In this paper, an automated segmentation method for low field 3D stomach MRI using transferred learning image enhancement network (TLLASN) is proposed. In the TLLASN model, our proposed multiscale 3D CycleGAN method is used to map the relationship between high and low field intensity MRI images, which overcomes domain shift between high and low field intensity images. And res-u-net is as the basic network, and then the convolution layer, batch normalization layer, and Relu layer together were replaced with a residual module. Furthermore, Dice loss function is selected to deal with the label sample unbalance in order to improve the segmentation ability of the proposed algorithm.

## 2. Dataset and Preprocessing

The experimental dataset included the low field MRI that were taken from 14 patients and the high field MRI that were taken from 9 patients in tumor radiation from the University of California, Los Angeles. The parameters of low field intensity MRI data acquisition are as follows: the thickness of scanning layer is 2-5 mm, the resolution of each scanning layer is 1.5 × 1.5 mm^2^, and the magnetic field intensity of scanning surface is 0.35 T. The parameters of high field intensity MRI data acquisition are as follows: the thickness of scanning layer is 2-5 mm, the resolution of each scanning layer is 1.5 × 1.5 mm^2^, and the magnetic field intensity of scanning surface is 3 T. In order to ensure the accuracy of data labeling, all the data are labeled by a radiologist. We randomly selected the images of four groups of patients from the data of 14 patients as the test set, and the rest of the images as the training set for deep network training and selected 20% of the training set as the validation set.

**Figure 1:**
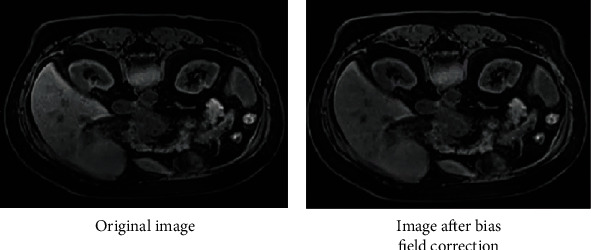
Image comparison before and after bias field correction.

In the course of magnetic resonance imaging, because of the change of magnetic field, MRI scan often shows intensity inhomogeneity, and the same tissue has also bright inhomogeneous change in vision. This change is called the bias field. Because the change of signal intensity is not due to anatomical differences, bias field will bring many problems to the subsequent image processing, which will aggravate the class imbalance and affect the image segmentation. Therefore, three preprocess strategies are made as follows:

(Step 1) Bias field correction: we use N4 bias field correction technology [13] to correct the image by extracting the bias field to ensure that each tissue type has the same intensity in a single image, as is shown in Figure 1.

(Step 2) Image resampling: all data were resampled with SimpleITK toolkit, and the resolution was uniformly sampled to 1.5 × 1.5 × 3.
(Step 3) Image cutting: (1) 2500 seed points were randomly scattered in the inner region of the whole 3D MRI image. (2) A 64 × 64 × 32 image patch is cut out centered on the selected point. (3) The label of each image is processed in the same way as (1) and (2). (4) Detect the number of pixels in the image patch cut out of the label one by one. If the number is more than 5, the image patch is retained; otherwise, the image patch and the corresponding MRI image patch are deleted together.
(Step 4) Data normalization: the gray value of the image needs be normalized to the [0, 1], when the full convolution neural network is used for image segmentation. The following normalization formula is taken on all images:

(1) $$\begin{array}{c} \hat{X}=\frac{X-\min\left(X\right)}{\max\left(X\right)-\min\left(X\right)}. \end{array}$$

## 3. Algorithm

The lack of low field intensity stomach MRI data and the large amount of 3D segmentation network parameters lead to overfit of the model. Zhang and his group have proved that the traditional data expansion is effective in reducing over fit and improving the generalization performance, especially without a large label training set. Therefore, expanding the existing low field MRI is the best way, such as rotating, flipping, and shearing the images. However, the traditional data enhancement methods lead to a high correlation between the expanded image and the original image, so the improvement of segmentation accuracy is limited. Another way of image expansion is to enhance the image by synthesizing the data with the same distribution as the target domain. The synthesized data does not come from the target domain directly and was obtained by a transferred image in different domains, which contains abundant anatomical and topological information, and is a good supplement to the target and image. Chartsias and his group [14] used CycleGAN to generate paired synthetic MRI and corresponding myocardial masks from paired CT slices and their myocardial segmentation masks. The authors based on CycleGAN image synthesis module, because it neither needs to match CT and MR image nor demands these images belonging to the same patient. Once the synthetic data was generated, synthetic MRI and original MRI are used to train the myocardial segmentation model and the segmentation performance increased 15% compared with the training of myocardial segmentation models only using real MRI.

Inspired by the study, the most direct solution is to enlarge the existing low field intensity MRI to improve the segmentation model. We expand training data by style transfer high field intensity MRI for network training in this paper, so that the trained model has good generalization ability. Cyclic Generation Adversarial Network can get the fake low field intensity MRI to map relationship between high and low field intensity MRI. Then, the fake low field intensity images generated by high field intensity magnetic resonance images through CycleGAN network are used as the extended data with transferred information. The low field MRI combines the extended dataset to train the 3D Res-Unet segmentation network, so as to overcome the problem of domain shift between high and low field intensity images.

### 3.1. Proposed Model

**Figure 2:**
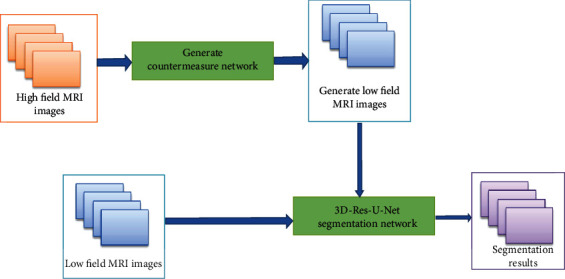
The architecture of the overall model.

This paper proposes a low field 3D MRI segmentation model based on transfer learning image enhancement, which is mainly composed of two parts: one is the high and low field intensity MRI image transfer network based on 3D CycleGAN model to map relationship, and the other is the stomach segmentation network based on 3D res-u-net. The model structure is shown in Figure 2.

### 3.2. Model Optimization

**Figure 3:**
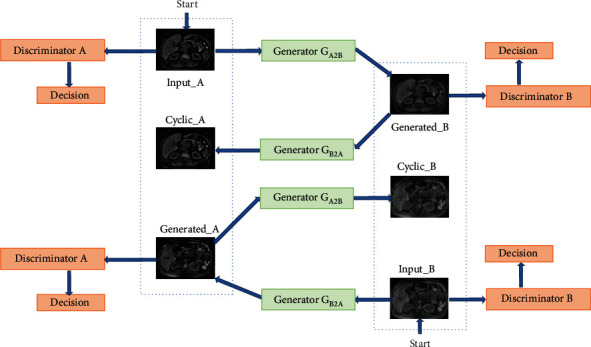
Structure diagram of high and low field intensity image transfer model.

The model structure of the 3D CycleGAN used is shown in Figure 3. There are five optimization strategies for the network to modify the traditional CycleGAN. First, two-dimensional convolution in CycleGAN is replaced by three-dimensional convolution. Secondly, in the generator part, the encoder decoder structure, i.e., the U-shaped structure, is adopted, and jump connection is added in the convolution layer corresponding to the encoder and decoder to fuse features of different scales. Thirdly, considering a certain correlation between the image patches of MRI, in the discriminator part, referring to the idea of PatchGan, the final output is 8 × 8 × 4 matrix, and then, the mean value of the matrix was calculated as true or false output. Fourthly, the stomach tag and MRI are used as the input of generator and discrimination, which makes the network pay more attention to the gastric region. Fifthly, label smoothing is used to reduce the label of real image from 1 to 0.9, which can avoid overconfidence of discriminator and improve the stability of model training.

**Figure 4:**
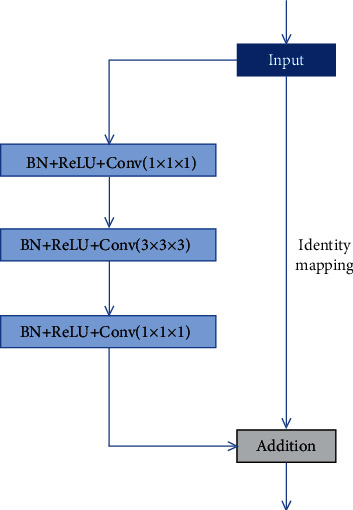
Bottleneck residual module.

There are three optimization to res-u-net the network. Firstly, the convolution layer, batch normalization layer, and Relu layer in 3D res-u-net are replaced together with a residual module, which increases the fitting ability of the network and alleviates the gradient disappearance of deep neural network. In order to reduce the parameters of the network without reducing the fitting ability of the network, a module with 1 × 1 × 1 convolution kernel is added before and after each convolution kernel is 3 × 3 × 3 modules, and its modified structure is shown in Figure 4.

Secondly, Dice loss is used as the loss function to solve the imbalance problem of positive and negative samples. The expression is rewrite as follows:

(2) $$\begin{array}{c} L_{\text{DC}}=1-\text{DC}\left(A,B\right). \end{array}$$

Thirdly, the convolution neural network generally requires that the input image size is fixed. For different size images, it is necessary to cut them to adapt to the input size of the network. In order to make the network adaptive to segmentation of any size images and reduce the memory consumption, the patch (64 × 64 × 32) is used to train the network here. In the test phase, the window is used to segment the image for patch prediction. The overlap between image patches is maintained (32 × 32 × 16), and the average value of the prediction results of overlapped parts is taken to reduce the patching effect and improve the accuracy of segmentation.

### 3.3. Evaluation Metrics

We used Dice coefficient (DC), sensitivity (SEN), specificity (SPE), Hausdorff distance (Haus), over segmentation rate (OR), and under segmentation rate (UR) to quantitatively analyze the segmentation results. Dice coefficient is used to measure the coincidence between the segmentation results and the gold standard. The larger the value, the higher the coincidence degree. It is more sensitive to the internal filling of segmentation results.

The calculation formula of Dice coefficient is as follows:

(3) $$\begin{array}{c} \text{DC}=2\times \frac{\left|X\cap Y\right|}{\left|X\cup Y\right|}. \end{array}$$

The sensitivity and specificity are calculated as follows:

(4) $$\begin{array}{c} \text{Sen}=\frac{\left|X\cap Y\right|}{Y},\\ \text{ Spe}=\frac{\left|X^{c}\cap Y^{c}\right|}{Y^{c}}. \end{array}$$

Hausdorff distance is the maximum distance between the segmentation result and the nearest point in the gold standard. The smaller the value is, the higher the similarity is, and it is more sensitive to the boundary of segmentation results. The formula is as follows:

(5) $$\begin{array}{c} \begin{array}{c} d_{H}\left(X,Y\right)=\max\left|d_{XY},d_{YX}\right|\\ =\max\left\{\max_{x\in X}\min_{y\in Y}d\left(x,y\right),\max_{y\in Y}\min_{x\in X}d\left(x,y\right)\right\}, \end{array} \end{array}$$

where *d*(*x*, *y*) is the Euclidean distance between the pixel in the segmentation result and the pixel in the gold standard.

The over segmentation ratio refers to the ratio of pixels whose segmentation results are beyond the gold standard. The calculation formula is as follows:

(6) $$\begin{array}{c} \text{OR}=\frac{O_{S}}{R_{S}+O_{S}}, \end{array}$$

where *O*_*S*_ is the number of pixels that should not have been included in the segmentation results, but actually are in the segmentation results, and *R*_*S*_ is the number of pixels in the segmentation results that coincide with the gold standard.

The under segmentation ratio refers to the ratio of pixels missing in the segmentation result within the gold standard. The calculation formula is as follows:

(7) $$\begin{array}{c} \text{UR}=\frac{U_{S}}{R_{S}+O_{S}}, \end{array}$$

where *U*_*S*_ is the number of pixels that should have been included in the segmentation results, but are not actually in the segmentation results.

### 3.4. Implementation Details

Our experiments were carried out using Keras with Ten-sorflow, whose backend is Python 3.5, and used Nvidia Ge-Force RTX2080, Cuda 9.0, and Cudnn v7.3.1 toolkit for parallel acceleration. The hardware configuration of the computer is 4.0 GHz Intel Core i7-4790k CPU. During optimization, Adam is used to optimize the generator and discriminator of cyclic generation countermeasure network. The weight of momentum is set to 0.5. The learning rate of the generator and discriminator is set to 0.0001 and 0.0004, respectively. In the segmentation network, the size of input image patch is 64 × 64 × 32, the batch size is set to 4, the optimizer uses Adam as the optimizer, the learning rate is 0.0001, and early stop is used to prevent over fitting.

## 4. Results

**Figure 5:**
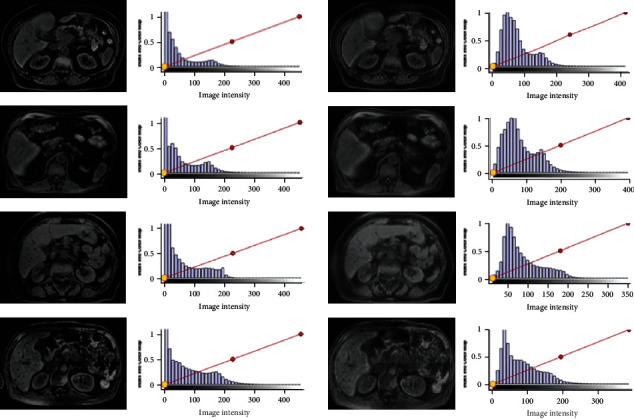
High field intensity MRI, axial image, and gray histogram of MRI were generated.

Figure 5 shows the axial and gray histogram of some high field MRI and the axial and gray histogram of the generated MRI images. The first column of each row is the axial image of the original high field intensity MRI, the second column is the gray histogram of the original high field intensity MRI, the third column is the fake image generated by CycleGAN, and the fourth column is the gray histogram corresponding to the transformed image. It can be seen that the histogram distribution of the transformed image is similar and follows the same distribution. From the perspective of anatomical structure, the position and shape of each organ in the image did not change, but the gray distribution was different, which is equivalent to the style migration, so the high field MRI label can be used as the label of the transferred image for segmentation network training.

**Figure 6:**
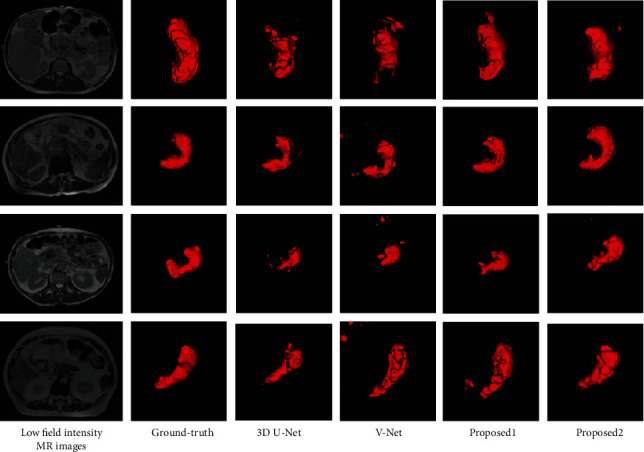
Image enhancement based on transfer learning in low field MR stomach segmentation.

In order to analyze the segmentation of different experiments more intuitively, the segmentation results are 3D reconstructed.  Figure 6 shows the low field MRI images and their 3D segmentation style of different patients using 4 deep networks, in which “Proposed1” means the segmentation method for traditional image enhance the low field intensity MRI by flipping and rotating and “Proposed2” means the segmentation method for traditional image enhance combining with transfer learning.

Compared with the segmentation results of 3D u-net and v-net, we can see that 3D u-net can segment the general stomach shape; however, under segmentation is more serious. V-net has improved to the under segmentation, but it is over segmentation. Compared with v-net and Proposed1, it can be seen that after the transfer learning image enhancement, the high field intensity MRI is transformed into the pseudo low field intensity MRI with the similar distribution as the low field intensity MRI by using CycleGAN, which increases the diversity of training samples and improves the segmentation performance significantly. Although “Proposed1” segmentation results also have partial over segmentation, the stomach region is smoother than that of 3D u-net and v-net. Compared with the results of the other three methods, “Proposed2” is a little over segmentation and under segmentation regions. To sum up, “Proposed2” has more completed region consistency and clear contour, which is closest to the ground truth.

Table 1 and Table 2 show the comparison of Dice coefficient and Hausdorff distance of segmentation results of different algorithms. From the point of view of Hausdorff distance, the indexes of different algorithms are almost the same, but the average Hausdorff distance of the proposed algorithm on the test set is optimal. From the perspective of Dice coefficient, there are different degrees of improvement in each test sample by using image enhancement strategy. The result of “Proposed2” shows that the use of high field intensity MRI transformed by CycleGAN network increases the diversity of training samples, alleviates the over fitting phenomenon to a certain extent, and improves the generalization ability of the network.

In order to analyze the under segmentation and over segmentation of 4 methods, we use indexes of sensitivity, specificity, over segmentation rate, and under segmentation rate for comparison, as shown in Tables 3–6. In Table 5, the over segmentation rate of the 4 methods is generally low, which shows that the over segmentation is not obvious in the segmentation results. This is consistent with the high specificity index in Table 4. The over segmentation rate of the algorithm we proposed is the lowest. Although the under segmentation rate of patient 3 in method 4 was slightly higher than that in method 2, its sensitivity was 1% higher. On the whole, the indexes of method 4 are better than those of the other three methods, which means that the segmentation model based on transfer learning image enhancement is effective for stomach segmentation of low field intensity MRI images.

Table 7 shows the comparison of segmentation results between the traditional data enhancement method and the combining image enhancement method. Traditional_based is to enhance the low field intensity MRI by flipping and rotating, CycleGAN_based is to enhance an image by using the trained CycleGAN network, which transformed the high field intensity MRI image into a pseudo low field intensity MRI image as the low field intensity MRI. From the perspective of segmentation index, each segmentation index of the CycleGAN_based is better than that of Traditional_based. Dice coefficient of CycleGAN_based is 2.5 percent higher than that of Traditional_based, over segmentation rate and less segmentation rate of CycleGAN_based are 0.7 and 5.5 lower percent than those of Traditional_based, respectively, and sensitivity of CycleGAN_based is higher 6.4 percent than that of Traditional_based.

## 5. Conclusion

The stomach is a kind of cavity organ in the abdomen, which is easy to deform and has uneven gray distribution. Moreover, low field intensity stomach MRI are noisy and lacking of data, which increases the difficulty of stomach segmentation in 3D images. TLLASN is proposed to cope with these problems. CycleGAN can get the fake low field intensity MRI to reduce the difference between high and low field intensity MRI. In other words, domain adaption between high and low field intensity images is achieved. In this study, the fake low field intensity images transferred information to train 3D Res-Unet segmentation network. High field intensity MRI is used to expand training data by style transfer for network training in this paper, so that the trained model has good generalization ability. The experimental results show that the automated segmentation method for low field 3D stomach MRI using transferred learning image enhancement network effectively increases the amount and diversity of training data and achieves good segmentation results.

## Figures and Tables

**Table 1:** Comparison of Dice coefficient (DC) index of four segmentation methods.

Method	#patient				Mean
	1	2	3	4	
3D U-net	0.562	0.704	0.648	0.349	0.566
V-net	0.613	0.759	0.597	0.493	0.616
Proposed1	0.684	0.865	0.681	0.532	0.690
Proposed2	0.654	0.874	0.730	0.631	0.722

**Table 2:** Comparison of Hausdorff distance index of four segmentation methods.

Method	#patient				Mean
	1	2	3	4	
3D U-net	10.05	10.1	7	7	8.54
V-net	9	8.1	6.71	8.60	8.10
Proposed1	9	7.87	6.71	8.60	8.04
Proposed2	9	7.87	6.4	8.60	7.97

**Table 3:** Sensitivity index comparison of four segmentation methods.

Method	#patient				Mean
	1	2	3	4	
3D U-net	0.395	0.568	0.495	0.212	0.417
V-net	0.472	0.697	0.539	0.396	0.526
Proposed1	0.535	0.811	0.551	0.509	0.602
Proposed2	0.497	0.814	0.600	0.465	0.594

**Table 4:** Comparison of specificity indexes of four segmentation methods in test set.

Method	#patient				Mean
	1	2	3	4	
3D U-net	0.999	0.999	0.999	0.999	0.999
V-net	0.999	0.999	0.999	0.999	0.999
Proposed1	0.999	0.999	0.999	0.999	0.999
Proposed2	0.999	0.999	0.999	0.999	0.999

**Table 5:** Comparison of over segmentation rate of four experimental methods in test set.

Method	#patient				Mean
	1	2	3	4	
3D U-net	0.011	0.044	0.031	0.003	0.023
V-net	0.063	0.122	0.211	0.172	0.142
Proposed1	0.029	0.064	0.064	0.025	0.045
Proposed2	0.023	0.044	0.043	0.008	0.029

**Table 6:** Comparison of four segmentation methods.

Method	#patient				Mean
	1	2	3	4	
3D U-net	0.598	0.413	0.489	0.785	0.571
V-net	0.494	0.266	0.364	0.499	0.406
Proposed1	0.491	0.180	0.382	0.530	0.396
Proposed2	0.451	0.174	0.419	0.478	0.381

**Table 7:** Comparison of segmentation results of different image enhancement methods.

Method	Metric	#patient				Mean
		1	2	3	4	
Traditional_based	DC	0.643	0.853	0.621	0.544	0.665
	Haus	9	8.062	6.708	8.485	8.064
	Sen	0.494	0.781	0.475	0.403	0.538
	Spe	0.999	0.999	0.999	0.999	0.999
	OR	0.039	0.047	0.052	0.073	0.053
	UR	0.486	0.208	0.497	0.553	0.436

CycleGAN_based	DC	0.684	0.865	0.681	0.532	0.690
	Haus	9	7.874	6.708	8	7.895
	Sen	0.535	0.814	0.551	0.509	0.602
	Spe	0.999	0.999	0.999	0.999	0.999
	OR	0.029	0.064	0.064	0.026	0.046
	UR	0.451	0.174	0.419	0.478	0.381

## Data Availability

Data used to support the findings of this study are included within the article
